# Pulmonary sclerosing hemangioma: a unique epithelial neoplasm of the lung (report of 26 cases)

**DOI:** 10.1186/1477-7819-11-85

**Published:** 2013-04-15

**Authors:** Bojiang Chen, Jun Gao, Hong Chen, Yidan Cao, Xin He, Wen Zhang, Man Luo, Shangfu Zhang, Weimin Li

**Affiliations:** 1 Department of Respiratory Medicine, West China Hospital of Sichuan University, No. 37, Guo Xue Street, Chengdu, Sichuan 610041, China; 2 Department of Laboratory of Stem Cell Biology, West China Hospital of Sichuan University, Chengdu, China; 3 Department of Toxicological Inspection, Sichuan Center for Disease Prevention and Control, Chengdu, China; 4 Department of Geriatric Medicine, Sichuan Academy of Medical Sciences & Sichuan Provincial People’s Hospital, Chengdu, China; 5 Department of Pathology, West China Hospital of Sichuan University, Chengdu, China

**Keywords:** Lung neoplasms, Sclerosing hemangioma, Immunohistochemistry, Thyroid transcription factor-1 (TTF-1), Epithelial membrane antigen (EMA), Cytokeratin (CK)

## Abstract

**Background:**

Pulmonary sclerosing hemangioma (SH) is an uncommon tumor. The aim of this study was to identify the origin of pulmonary SH and summarize its clinicopathologic features.

**Methods:**

Data of 26 cases of pulmonary SH were collected and reviewed, including their clinical symptoms, chest radiological examinations, treatments, and pathological findings.

**Results:**

Female patients of pulmonary SH were markedly frequent (*n*=23, 88.46%). Solitary mass or nodule in the lung fields was the most common manifestation (*n*=24, 92.31%), especially in the right middle lobe (*n*=9, 34.62%). There were two kinds of tumor cells: lining cells and round cells. All tumors contained a mixture of papillary, solid, sclerotic, and hemorrhagic patterns. Immunohistochemistry with a variable number of antibodies was performed for some cases. All of the detected specimens revealed strong reaction of lining cells with epithelial markers, such as thyroid transcription factor-1 (TTF-1), epithelial membrane antigen (EMA), cytokeratin (CK), pancytokeratin (PCK), and cytokeratin 7 (CK-7), while round cells were positive with TTF-1 and EMA. Until the end of last contact, none of the patients died or suffered from the recurrence of the disease after surgical treatment.

**Conclusions:**

Pulmonary SH is a unique neoplasm of the lung with a characteristic solitary mass or nodule. Pulmonary epithelium might be the primary origin of the tumor cells.

## Background

Pulmonary sclerosing hemangioma (SH) is a rare neoplasm, which was described by Liebow and Hubbell over 50 years ago [1]. Though it was generally regarded as a benign tumor, its histogenesis and biologic behaviors is still a controversy [2-7]. Additionally, notwithstanding the original term of ‘sclerosing hemangioma’, more and more evidence suggested it might not be derived from vascular-endothelium [4-6]. Here, we reviewed 26 cases of pulmonary SH patients, including their clinical manifestations, radiological findings, treatments, and pathological results, to explore and summarize its characteristics.

## Methods

### Patients and tissue samples

A total of 26 surgically resected and histopathological examination confirmed pulmonary SH cases, in the Department of Thoracic and Cardiovascular Surgery, West China Hospital of Sichuan University, China, during the period January 2009 to May 2012, were recruited to this study. Clinical data, such as symptoms, signs, radiological examinations, and treatments, were reviewed from the patients’ charts. Institutional review board approval for the research was obtained from the Ethics Committee of Sichuan University. Consent was obtained from all patients.

### Immunohistochemical staining

All tumor tissues were embedded in the paraffin blocks after being fixed by 4% neutral formaldehyde, and the following slices were approximately 4 μm. Hematoxylin-eosin stainings were the basis for the diagnosis. Further immunohistochemical stainings were performed on tumor tissues from 13 subjects using the avidin-biotin method to confirm the origin of the tumors. The mouse monoclonal antibodies included: epithelial membrane antigen (EMA, Dako (Dako North America Inc., CA, USA), 1:100), pancytokeratin (PCK, Dako, 1:100), cytokeratin (CK, Dako, 1:100), cytokeratin 7 (CK-7, Dako, 1:100), thyroid transcription factor-1 (TTF-1, Dako, 1:100), desmin (Dako, 1:100), synaptophysin (Syn, Dako, 1:100), chromogranin (CgA, Dako, 1:100), S-100 protein (Dako, 1:100), neuron-specific enolase (NSE, Dako, 1:100), and Ki-67 (Dako, 1:100). Deparaffinized sections were treated with methanol containing 3% hydrogen peroxide for 10 min before conducting antigen retrieval using a microwave oven at 95°C for 5 min and cooling at 25°C for 2 h. The sections were incubated with target antibodies overnight at 4°C. After washing with phosphate buffer saline (PBS), a biotin-marked secondary antibody was applied for 10 min followed by a peroxidase-marked streptavidin for an additional 10 min. The reaction was visualized by using 3, 3’-diaminobenzidine tetrahydrochloride. Finally, the sections were counterstained with Harris hematoxylin, dehydrated and cleared in xylene, and mounted [8]. External or internal tissues known to express the target proteins were used as positive controls, while negative controls referred to those whose primary antibodies were replaced by PBS.

### Immunohistochemical assessment

Expression of each marker was assessed semi-quantitatively according to the criteria that accounted for both the fraction and intensity of immunostaining of tumor cells involved. The fraction score was defined as the average of five randomly selected fields in light microscope: 0, no cell stained; 1, <20% of cells stained; 2, 20% to 50% of cells stained; and 3, >50% of cells stained. The intensity scores were defined as follows: 0, no appreciable staining in the tumor cells; 1, barely detectable staining in the cytoplasm and/or nucleus compared with the stromal elements; 2, readily appreciable brown staining; and 3, dark brown staining in tumor cells obscuring the cytoplasm and/or nucleus. The total score was calculated by multiplying the fraction score and the intensity score producing a total range from 0 to 9. For the final analysis, total scores of 0 to 2 were considered negative/low expression, and scores of 2 to 9 positive/high expression [9,10]. All histopathological evaluation was carried out by two board-certified pathologists with more than 5 years of experience.

## Results

### Clinical characteristics

Details of the clinical characteristics of the study cohort are summarized in Table 1. Among the 26 cases, there were 23 women and only 3 men, indicating a female:male ratio of 7.67:1. The patients ranged in age from 31 to 68 years, with a median age of 46 years. The most frequent symptoms were cough and sputum (*n*=11, 42.31%). Eight patients (30.77%) had hemoptysis, seven of whom were characterized by repeated bloody sputum, whereas the remaining patient was spitting up full of blood. However, another nine patients (34.62%) were reported to have lesions accidentally found by routine chest X-ray examinations without any discomfort. Nobody had a positive chest sign in physical examinations. As for past medical histories, only one 64-year old female patient had smoked for >20 years, nearly six cigarettes per day.

**Table 1:** Clinical characteristics of 26 pulmonary SH patients

**Characteristics**	**Cases ( *n *)**	**Characteristics**	**Cases ( *n *)**
*Symptoms*		*Lesion location*	
Cough	11	Left upper lobe	3
Sputum	11	Left lower lobe	5
Hemoptysis	8	Right middle lobe	9
Dyspnea	4	Right left lobe	6
Chest pain	4	Right middle and lower mediastinal	1
Asymptomatic	9	Left upper and lower lobes	1
*Characteristics on imaging examinations*		Bilateral lower lobes	1
Mass	17	*Intraoperative frozen biopsy*	
Nodule	9	Pulmonary SH	20
*Enhanced CT scans*		Hamartoma	1
Not done	15	Adenocarcinoma	3
Mildly enhanced	5	Bronchioloalveolar carcinoma	1
Significantly enhanced	1	Adenosquamous carcinoma	1
Heterogeneously enhanced	2		
Homogeneously enhanced	1		
No enhancement	2		

**Figure 1:**
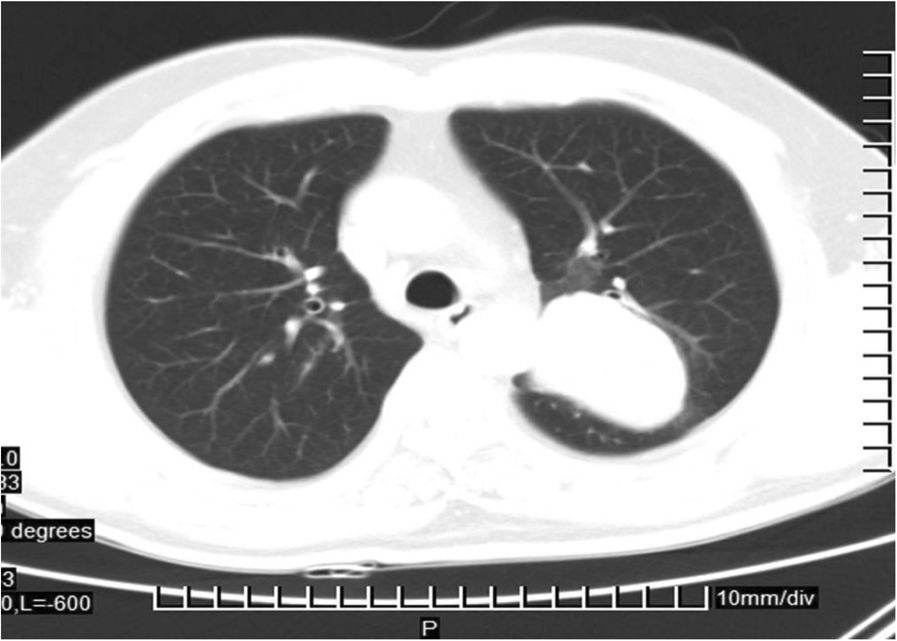
One mass of approximately 58.2 mm in the left lung with a smooth margin.

All patients had chest computed tomography (CT) scans. Most tumors (*n*=15, 57.69%) were located in the right lung, especially in the middle lobe (*n*=9, 34.62%). The typical radiological appearance was a solitary nodule (9 cases, with diameters >20 mm) or mass (17 cases, with diameters >20 mm). However, in one patient, there were multiple distinct nodules in the bilateral lower lobes. Another case was presented with two masses in the upper and lower lobes of the left lung, but only the latter was confirmed with pulmonary SH in the following pathological examination. A right middle and lower mediastinal mass was unique. The mean long-axis diameter for the SH lesions was 29.23 mm, ranging from 10 mm to 72 mm. Apart from one lesion with an irregular shape, two had fuzzy boundaries, and another one with short spiculation, the remaining 22 cases (84.62%) had round or round-like tumors with smooth margins (Figure 1). Compared with multiple spots and flake calcification in two masses, the internal texture for the remaining lesions on normal CT scans was homogeneous, though there was some marginal calcification in one mass. Lymph node enlargement in the left hilar was found in another case. Enhanced CT scans were administrated for 11 patients, only two (2/11=18.18%) without any enhancement.

In order to make an accurate diagnosis, 15 patients received fiberoptic bronchosocopy examinations before surgeries, but they all indicated chronic inflammation without evidence of tumors.

According to the existing clinical data mentioned above, only one patient was diagnosed with pulmonary SH; the other initial diagnoses were lung cancer (*n*=10, 38.46%), tuberculoma (*n*=4, 15.38%), inflammatory nodule (*n*=1, 3.85%), other benign lesions (*n*=7, 26.92%), and undetermined (*n*=3, 11.54%).

Surgeries were administrated for all patients, and video-assisted thoracoscopic surgery (VATS) was the most common procedure (*n*=16, 61.54%), in particular VATS lobectomy (*n*=13, 50.00%). No marked pleural adhesion or effusion was detected. Lesions were tough and well enveloped by capsules, without apparent invasions. In addition to one mass in the right middle and lower mediastinum, the other 25 masses were located in the peripheral lung fields. Because of the erroneous intraoperative frozen biopsy diagnosis, four subjects underwent lymph node dissection. Another one had an adenosquamous carcinoma in the left upper lobe and pulmonary SH in the left lower lobe at the same time, but the latter was a missed diagnosis until the postoperative histopathological examination. All patients were alive without any significant complications by the last contact. The patient with adenosquamous carcinoma has been receiving chemotherapy.

### Histopathological characteristics

#### Gross features

Gross descriptions were available for the whole specimens. The maximum diameters of SH lesions varied from 5 mm to 70 mm, average 26.85 mm. Five cases (19.23%) were >10 mm, while another four (15.38%) were ≥50 mm. All SH masses or nodules were well-circumscribed and could be shelled out easily from the surrounding pulmonary tissues. The tumors’ cut surfaces presented as granular, rubbery, or meaty, coloring from gray-tan, yellow to dark red.

#### Histologic features

Lesions were mainly composed of varying proportions of two types of cells. The first one was epithelial cells, which resembled type II alveolar pneumocytes. They were coating on the surface of tumors, or the inner surface of cavities, with morphologies of cubic, flat, oval, or cylindrical appearance, called ‘lining cells’. The second cluster was the underlayer interstitial mononuclear cells. They are distributed in the interstitium, characterized by uniform, medium size, and are oval or polygonal in shape. Clear or pale staining cytoplasm, predominantly oval nuclei with fine and evenly distributing chromatin, occasional small nucleoli without nuclear division or necrosis were frequently seen. They were described as ‘round cells’.

**Figure 2:**
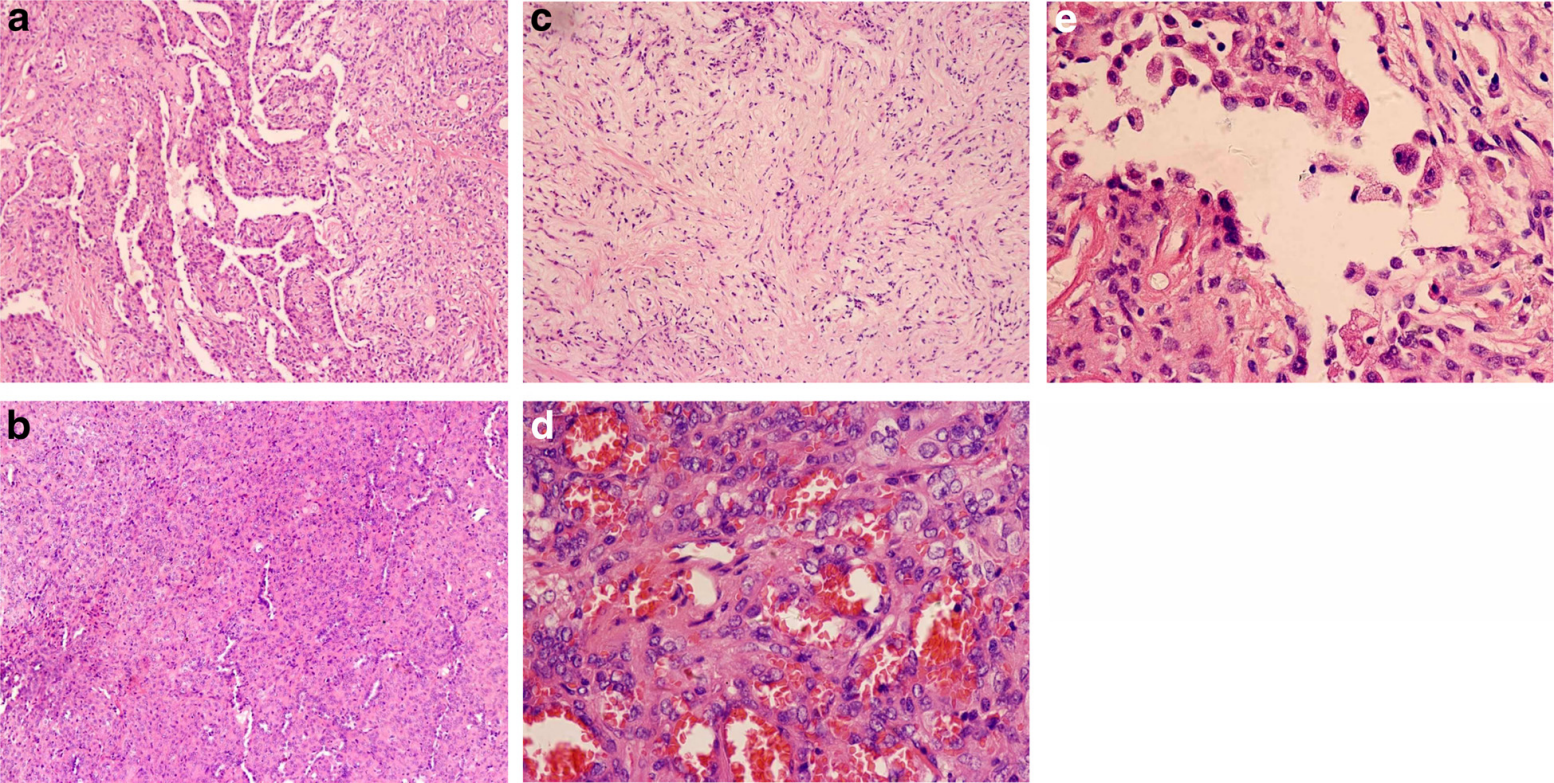
**Four major histologic patterns of pulmonary SH by hematoxylin-eosin stains.** Pulmonary SH showed papillary (**a**, ×100), solid (**b**, ×100), sclerotic pattern (**c**, ×100), and hemorrhagic (**d**, ×400). (**e**) Lining cuboidal cells and stromal round cells (×400).

According to the number of tumor cells and tissue morphological features, lesions included four major histological patterns: papillary, sclerotic, solid, and hemorrhagic (Figure 2). The papillary pattern was usually lined by small uniform round or cuboidal cells, type II pneumocytes, and bronchiolar epithelium, presenting as papillary appearance instilled into the alveolar spaces or the surface of tumors. There were different degrees of proliferation in the lining cells, even atypia or forming stratified structures. The cuboidal cells often demonstrated multinucleation and intranuclear inclusions, sometimes with vacuolated cytoplasm. Other different morphologies between the round cells and the surface lining cells could be clear. For example, hemosiderin was seen in the surface lining cell in two cases, but the underlying round cells were free of such particles.

Sclerotic foci were formed by dense interstitial fibrosis and hyaline collagen tissues in the alveolar intervals, often adjacent to hemorrhagic areas or solid zones. Small sclerosed blood vessels and scattered round tumor cells were easily found.

The solid pattern was composed of sheets of uniform round to polygonal cells with round or oval nuclei, which were centrally located, but mitotic figures were rare. The round cells were lined by cuboidal cells with smaller and darker nuclei. They demonstrated entrapped alveolar spaces with residual reactive alveolar lining cells. Focally accumulated foam cells, singly or clusteringly distributed erythrocytes were also commonly interspersed around the round cells to form small blood lakes.

Hemorrhagic lesions were filled with vascular-like channels or true vasculars. They were large, irregular, and dilated, packed with varying amounts of erythrocytes. A single layer of cuboidal cells or flat cells such as endothelium were lined in the walls of channels, some of which had hyalinosis. Typical round cells were always in the interstitium.

Among the 26 cases, all tumors composed of more than one histologic pattern. The majority (*n*=15, 57.69%) demonstrated a variable proportion of the whole four patterns. Nine tumors (34.62%) were composed of three patterns, including papillary, sclerotic, and solid (*n*=5, 19.23%), papillary, sclerotic, and hemorrhagic (*n*=2, 7.69%) and the remaining two were papillary, hemorrhagic, and solid, and hemorrhagic, sclerotic and solid, respectively. The additional two cases (7.69%) contained a mixture of two patterns: papillary with sclerotic for one subject and papillary with hemorrhagic for the rest.

All pathological findings, including the additional features, are listed in Table 2. Focal chronic inflammation, such as interstitial fibrosis and lymphocytic infiltration, was displayed in all cases. Hemosiderin in the alveolar spaces or interstitium was a common appearance. Another distinctive feature of 17 cases was the varying degrees of atypia, leading to an erroneous intraoperative frozen biopsy diagnosis of adenocarcinoma, adenosquamous carcinoma, and bronchioloalveolar carcinoma in four cases.

**Table 2:** Histological features of the 26 cases of pulmonary SH

**Histological pattern**	***n***
Papillary	25
Sclerotic	24
Solid	22
Hemorrhagic	20
Chronic inflammation	26
Mast cells	23
Hemosiderin	22
Atypia	17
Mild	13
Moderate	4
Eosinophils	10
Calcification	8
Cholesterol clefts	6
Lamellar structures	5
Necrosis	2
Hyaline degeneration	1
Adenosquamous carcinoma	1

We experienced one unusual case with more than one nodule and masses in the left lower lobe with diameters from 8 mm to 30 mm. Only the largest one was detected on CT scans and identified as adenocarcinoma in the intraoperative frozen biopsy. However, further examination confirmed the pulmonary SH for the small nodule of approximately 8 mm and other lesions. Moreover, another subject held two masses in the left lower and upper lobes. Pathological evidence revealed the former one was pulmonary SH, rather than the metastatic lesion for the adenosquamous carcinoma in the upper lobe, which was diagnosed from clinical information.

Fortunately, no lymph nodule was detected with pulmonary SH metastasis.

### Immunohistochemistry

Thirteen cases underwent immunohistochemistry with a variable number of antibodies to determine the phenotype of tumor cells. The results are shown in Table 3.

**Table 3:** Immunohistochemical findings of 13 cases of pulmonary SH

**Markers**	**Antibodies**	**Cases examined ( *n *)**	**Lining cells (*n***^**a**^**, %**^**b**^**)**	**Round cells (*n***^**a**^**, %**^**b**^**)**
Epithelial markers	EMA	12	12 (100.00%)	12 (100.00%)
	PCK	7	7 (100.00%)	0 (0.00%)
	CK	5	5 (100.00%)	0 (0.00%)
	CK-7	8	8 (100.00%)	2 (25.00%)
	TTF-1	10	10 (100.00%)	10 (100.00%)
Neuroendocrine markers	Syn	7	0 (0.00%)	0 (0.00%)
	CgA	6	0 (0.00%)	0 (0.00%)
	S-100	6	0 (0.00%)	0 (0.00%)
	NSE	3	0 (0.00%)	0 (0.00%)
Vascular-endothelial markers	CD34	3	1 (33.33%)	0 (0.00%)
Myoepithelial markers	Desmin	1	0 (0.00%)	0 (0.00%)
Proliferation markers	Ki-67	4	0 (0.00%)	4 (100.00%)^c^

^a^Number of cases revealing positive results.

^b^Proportion of positive cases to the whole examined ones.

^c^The expression of Ki-67 in all positive cases was <5%.

**Figure 3:**
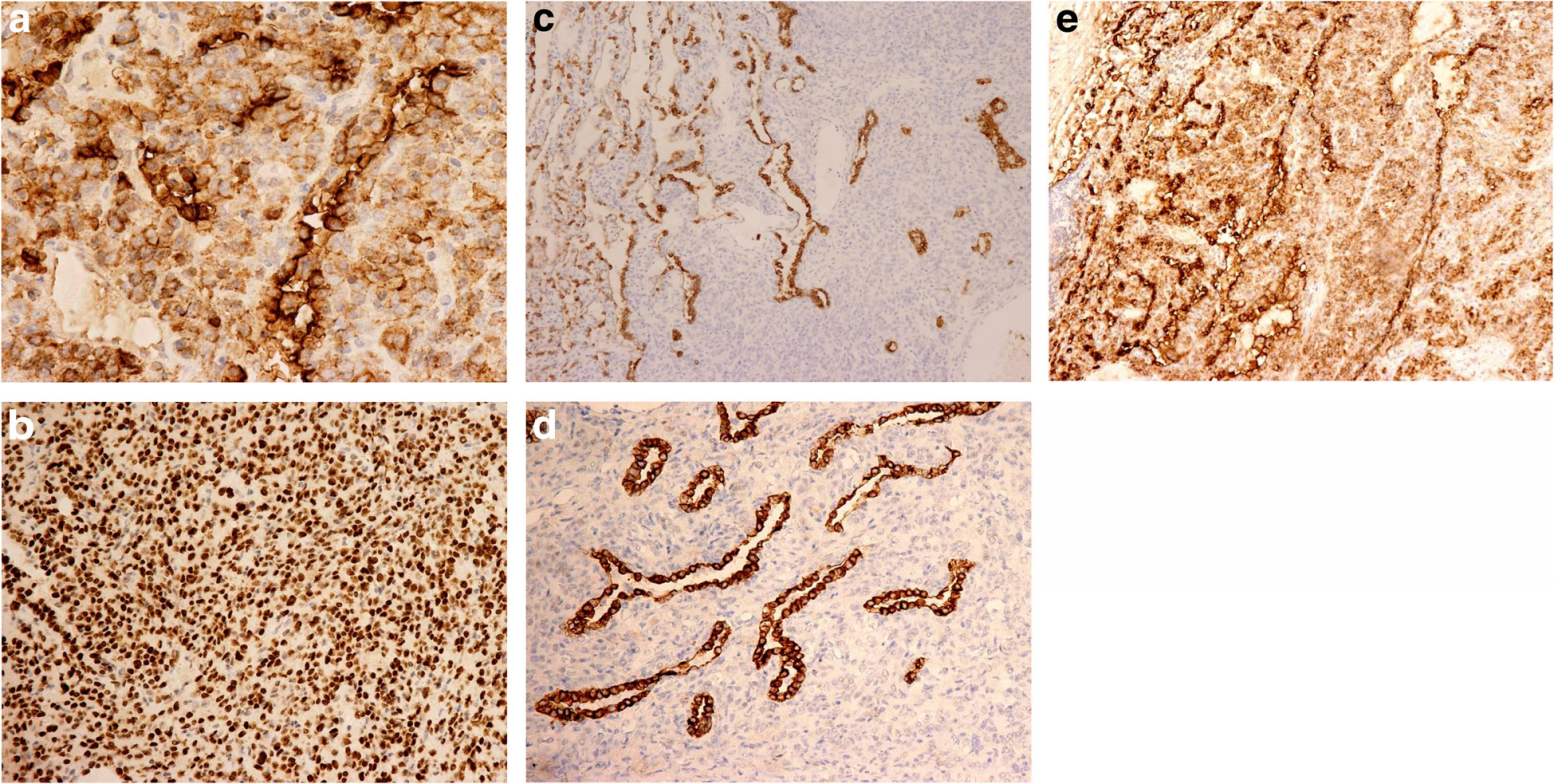
**Immunohistochemistry of pulmonary SH by the labeled streptavidin-biotin peroxidase technique.** Immunohistochemical stains showed both lining cells and round cells were positive for EMA (**a**, ×400) and TTF-1 (**b**, ×200). The lining cells were positive for CK (**c**, ×200), CK-7 (**d**, ×200) and PCK (**e**, ×200).

Both surface lining cells and round cells were diffusedly strongly stained with EMA (100.00%). The lining cells were in a cytoplasmic positivity, and the round cells displayed the main immuoreaction in membrane. Compared with negative reaction for round cells with PCK (0.00%), CK (0.00%), and CK-7 (25.00%), stains of PCK, CK, CK-7, EMA, and TTF-1 were completely positive (100.00%) in all tested lining cells without exception (Figure 3). Although the nuclear stains of TTF-1 in round cells were a little weaker than those of lining cells, they were all met with the positive standard, and in focal to extensive stains. Nevertheless the number of cases stained by the neuroendocrine markers of Syn, CgA, S-100 and NSE, and myoepithelial markers of desmin was limited, no positive finding in all tumor cells was still a meaningful result. One case with a strong appearance of EMA test exhibited a faint detection of the vascular-endothelial marker of CD 34, but the negative trend of CD 34 staining in other specimens was still significant. In spite of the positive expression of Ki-67 in round cells in four tumors, it was extremely rare to scattered but never numerous. The positive proportion only ranged from 1% to 5%. Moreover, it failed to stain the all lining cells.

## Discussion

Pulmonary SH is an uncommon, but histologically distinctive neoplasm of the lung. Although several researches have been conducted for its morphology, the histogenesis is still contested. The most meaningful finding of the current immunohistochemical study was the completed positive reaction of EMA and TTF-1 both with the lining cells and round cells. Other epithelial markers, such as CK, CK-7 and PCK, also disclosed full stains with the lining cells. The consistent outcomes with previous reports [2-4] not only allowed us to speculate that epithelial cells were the origin of pulmonary SH tumor cells, but also indicated these antibodies were effective approaches for the diagnosis of this lesion.

TTF-1, also known as Nkx2.1, is a 38-kd homeodomain containing DNA-binding protein expressing in the thyroid epithelium, ventral forebrain, and bronchial epithelial cells [11]. It is also essential for determining the pulmonary morphogenesis and gene expression [12]. Currently, TTF-1 has been widely used as the specific marker for type II pneumocyte in studies of respiratory cancers. The positive result of TTF-1 in this cohort of tumors suggests that pulmonary SH is probably derived from alveolar epithelium. TTF-1 in the lung also regulates the surfactant proteins, which are a family of important innate immunity effectors and critical for lung stability and lung host defenses [11]. Co-expression of TTF-1 and surfactant proteins is usually detected in health adult bronchioles, alveolar epithelum, and well-differentiated differentiated adenocarcinoma [13].

EMA is generally assumed to be expressed only on a wide variety of human non-squamous epithelial surfaces [14-16]. CK is another kind of scaffold protein in epithelial cells. The expression of CK always can be detected in the whole process of cells differentiation or even carcinogenesis. In particular, it contains series of members with different molecular weights from 40 KD to 70 KD, each of them suggesting a specific feature for the target cells. In this study, apart from two cases with positive reaction of CK-7 in round cells, other round cells revealed uniformly negative expression of PCK and CK. However, lining cells were strongly immunoreacted with CK, PCK, and CK-7. This immunophenotype provides insights into the further possible discrepant cellular differentiation between the two kinds of cells. Lining cells appears to be more differentiated than round ones. A microdissection study also demonstrated the similar clonality of lining cells and round cells in pulmonary SH, but they represented a variable differentiation from the same progenitor cell [17]. Furthermore, the lack reaction of Syn, CgA, S-100, NSE, CD34, and Desmin supported the origin of tumor cells from pulmonary epithelium, rather than neuroendocrine, vascular-endothelium, or myoepithelium.

Although pulmonary SH is regarded as a benign tumor, rare cases revealed metastasis, especially involving mediastinal or hilar lymph nodules [18-20]. However, the distant hematogenous metastasis has never been reported until now. Kim and colleagues [20] assumed that the larger size of primary tumors and male patients were more likely to have metastasis, which has not been proved by sufficient statistical data yet. But the follow-up information for cases over 3 years showed no apparent effects on the prognosis [21-24]. In this study, we found no metastatic cases after complete excision of tumors. In spite of inadequate follow-up intervals, it was probably a piece of evidence for the inactive biologic behaviors of pulmonary SH. Besides, the expression of Ki-67 in lining cells was completely negative, and for the round cells, only <5% ones weakly expressed it as well.

To a large extent, lesions of pulmonary SH are generally a solitary mass. Multiple or bilateral lesions were rarely described [25,26] and the possible pathogenesis was unclear. In our series, there were two cases with more than one mass on CT scans. Pathological examinations confirmed one patient suffered from adenosquamous carcinoma in the left upper lobe and pulmonary SH in the lower lobe, whereas the other one had bilateral lesions in both of the lung fields. We did not investigate whether this was a multicentric origin of pulmonary SH case or intrapulmonary metastasis. Moreover, atypical alveolar hyperplasia (AAH) is considered to be associated with the incidence of multiple SH lesions [21]. The specific mechanism is needed to be explored.

Similarly to pulmonary SH, bronchioloalveolar carcinoma (BAC), a subtype of pulmonary adenocarcinoma, is another tumor derived from alveolar epithelial cells, which has been confirmed by molecular genetic techniques already [27]. The differential diagnosis of pulmonary SH and BAC is important. Based on its pathological features, there are two types of BAC, the mucinous tumor and non-mucinous tumor. The former is characterized by mucus cells and mucus lakes, leading an easy pathological identification, while the tumor cells for non-mucinous BAC show an alveolar cell differentiation. However, larger cell sizes, with deeper nuclear chromatin stainings, much more marked pleomorphism and even pathologic mitosis, makes BAC different from pulmonary SH. For atypical cases of pulmonary SH, positive expression of EMA drawing the outline tumors cells helps to confirm the diagnosis as well. Also, positive stainings with antibodies of cyclinD1 and eukaryotic initiation factor 4E (eIF4E) have been recommended for the diagnosis of BAC [28].

As a pulmonary neoplasm, SH is frequently presented in the lung fields. Unusual cases of extralobar pulmonary SH have been reported by Ahmetoglu [26] and Sakamoto [29]. There were three hypotheses to explain these findings [21,26]. The first one was that the extrapulmonary lesions, such as mediastinal masses, were metastatic foci of the primary pulmonary lesions. Second, tumors might derive from the ectopic lung tissues. Third, the SH developed as a pedunculated pleural mass from the lung and moved towards the lung surface or mediastinum. But none of them has been confirmed. We discovered one patient with a mass of approximately 54 mm with heterogeneously enhancement on enhanced CT scans and right atrium was extrusion. The initial diagnosis was the usual lung cancer or other mediastinal tumors. Intraoperative frozen examination identified the diagnosis of pulmonary SH. Following careful reviews of the chest radiological images revealed that the tumor arose from the periphery of the lung and developed into the central of mediastinum, giving a false impression of lung cancer or mediastinal neoplasm.

## Conclusion

In conclusion, pulmonary SH is a unique neoplasm of the lung. Solitary masses or nodules in the lung fields, especially in the right middle lobes, without apparent metastatic lesions, are the most common radiology manifestation. Surgical resection can obtain histopathological specimens and cure the disease. The tumor cells include typical superficial lining cells and underlying round cells, which might origin from the pulmonary epithelial cells, characterized by the positive staining of epithelial markers, such as EMA and TTF-1. But the pathological mechanisms are needed to be further elucidation.

## Competing interests

The authors declare that they have no competing interests.

## Authors’ contributions

BC, JG, and HC carried out data collection of all patients, including clinical information and pathological diagnosis, performed the statistical analysis and the manuscript writing. YC, XH, WZ, and ML participated in the immunohistochemistral staining and scoring. SZ and WL participated in the whole study design and coordination and helped to revise the manuscript. All authors read and approved the final manuscript.
